# Impact of primary breast cancer therapy on energetic capacity and body composition

**DOI:** 10.1007/s10549-018-4924-6

**Published:** 2018-08-22

**Authors:** Tarah J. Ballinger, Anurag Reddy, Sandra K. Althouse, Emily M. Nelson, Kathy D. Miller, Jeffrey S. Sledge

**Affiliations:** 1 Indiana University School of Medicine, Indianapolis, IN USA; 2 Department of Urban and Regional Planning, University of Wisconsin, Madison, WI USA; 3 Division of Hematology/Oncology, Department of Medicine, Indiana University School of Medicine, Indianapolis, IN 46202 USA

**Keywords:** Breast cancer, Physical activity, Power, Body composition, Energetic capacity, Sarcopenia

## Abstract

**Purpose:**

This observational study was designed to measure baseline energy parameters and body composition in early-stage breast cancer patients, and to follow changes during and after various modalities of treatment. This will provide information to aid in the development of individualized physical activity intervention strategies.

**Methods:**

Patients with newly diagnosed stage 0–III breast cancer were enrolled into three cohorts: A (local therapy alone), B (endocrine therapy), or C (chemotherapy with or without endocrine therapy). At baseline, 6 months, and 12 months, subjects underwent a stationary bicycle protocol to assess power generation and DEXA to assess body composition.

**Results:**

Eighty-three patients enrolled. Patients had low and variable levels of power generation at baseline (mean power per kilogram lean mass 1.55 W/kg, SD 0.88). Power normalized to lean body mass (W/kg) decreased significantly, and similarly, by 6 months in cohorts B (1.42–1.04 W/kg, *p* = 0.008) and C (1.53–1.18 W/kg, *p* < 0.001). In all cohorts, there was no recovery of power generation by 12 months. Cohort C lost lean body mass (− 1.5 kg, *p* = 0.007), while cohort B maintained lean body mass (− 0.2 kg, *p* = 0.68), despite a similar trajectory in loss of power. Seven patients developed sarcopenia during the study period, including four patients who did not receive any chemotherapy (cohort B).

**Conclusions:**

The stationary bike protocol was feasible, easy, and acceptable to patients as a way to measure energetic capacity in a clinical setting. Early-stage breast cancer patients had low and variable levels of power generation, which worsened following primary therapy and did not show evidence of ‘spontaneous recovery’ by 12 months. Effective physical activity interventions will need to be personalized, accounting for both baseline ability and the effect of treatment.

**Electronic supplementary material:**

The online version of this article (10.1007/s10549-018-4924-6) contains supplementary material, which is available to authorized users.

## Background

An accumulating body of evidence supports an association between physical activity and improved quality of life, overall health, and disease-related outcomes for millions of breast cancer survivors. A large meta-analysis of observational studies found a significantly decreased risk of all-cause and breast cancer-specific mortality for survivors participating in higher levels of post-diagnosis physical activity, compared with lower levels (HR 0.52, 95% CI 0.43–0.64, *p* < 0.01; and HR 0.59, 95% CI 0.45–0.78, *p* < 0.05, respectively) [1]. Unfortunately, the majority of breast cancer survivors are physically inactive and self-report stable or decreased physical activity levels following diagnosis [2, 3].

Exercise intervention trials for breast cancer survivors are feasible and result in beneficial effects on body composition, fitness, fatigue, and quality of life [4, 5]. However, only a fraction of eligible patients agree to participate and even fewer complete the prescribed intervention, limiting the generalizability of these interventions. Patients who report more fatigue or who have limited prior exercise experience are less likely to participate or comply; consequently the precise population in greatest need is the least likely to benefit from current approaches [6–8].

Breast cancer patients, like the general population, have variable levels of exercise capacity both at the time of diagnosis and post- treatment. Although generic, population-based guidelines for physical activity in cancer survivors exist, these are not well defined and are not tailored to a patient’s starting exercise capacity [9].Too often, these guidelines may not be initially achievable for individual breast cancer patients, leading to frustration, injury, early discontinuation, or opting out of exercise interventions entirely. In addition, while patients may subjectively describe fatigue or weakness, clinically feasible objective measures of exercise capacity to guide interventions and construct individualized recommendations are lacking.

This observational study was conducted to provide the foundational data needed to develop individualized physical activity intervention strategies for early-stage breast cancer patients. We sought to measure baseline energy parameters and body composition, and to follow changes in those parameters during treatment. Evaluation 1 year from diagnosis was included to assess ‘spontaneous recovery’ after finishing treatment.

## Methods

### Study population

This prospective study enrolled patients with newly diagnosed ductal carcinoma in situ (DCIS) or stage I–III invasive breast cancer presenting to the Indiana University Melvin and Bren Simon Cancer Center and Eskenazi Health in Indianapolis, Indiana. Eligible patients had not yet initiated any therapy for their breast cancer and had no neurologic, orthopedic, cardiac, or pulmonary conditions that would interfere with the ability to complete the stationary bicycle protocol. Patients were enrolled into one of the three cohorts based on planned therapy: cohort A included patients who would receive local therapy alone (surgery ± radiation therapy), cohort B included patients planned to receive local therapy with anti-estrogen therapy as the sole systemic treatment, and cohort C included patients planned to receive local therapy and chemotherapy with or without anti-estrogen treatment. Planned treatment was indicated at the time of study registration. Patients registered to cohorts A and B who received chemotherapy based on findings at the time of surgery were transferred to and analyzed with cohort C. Patients were compensated for the time required to complete the study assessments. The IU Institutional Review Board approved the study and patients provided written informed consent prior to participation. Research has been performed in accordance with the ethical standards of the Declaration of Helsinki.

### Study design

#### Energetic capacity

The primary objective was to establish energetic capacity. Ten seconds mean peak watts (watts) and watts per kilogram (kg) of lean body mass (watts/kg) of newly diagnosed breast cancer patients were recorded at baseline and at 6- and 12-month post-diagnosis. We quantified energetic capacity using the Power Protocol-B™, a stationary bicycle-based procedure that establishes the range of motive power performance of the patient (termed “power envelope”). The Power Protocol-B™ is less invasive, costly, intimidating, and technically difficult than other standard measures of fitness, such as maximal VO_2_, and can be performed in an ambulatory clinic setting [10]. Measures obtained from Power Protocol-B™ include, but are not limited to, minimal and maximal power production, sustained power at a given heart rate, and heart rate separation zone, which is one indicator of anaerobic threshold. Importantly, data generated from Power Protocol-B™ are independent of patient effort over a wide range of ability.

Power Protocol-B™ testing was conducted on a Saris CycleOps 400 Pro stationary bicycle (Saris cycling Group, Fitchburg, WI) incorporating a 45-pound flywheel to smooth power pulses from rider impulses to the cranks. PowerTap resistive strain gauge sensors in the wheel measure power (watts) with a validated accuracy and linearity of 98.5% [11]. The 400 Pro is equipped with a wireless central processing unit (CPU), a wireless heart rate strap (recording directly to an integrated data file in the CPU), and a configurable display set to display power, cadence, derived speed, heart rate, and elapsed time. The bicycle readily accommodates a wide variety of body types; patients were individually fit before the initial assessment using accepted biodynamic fit procedures. Patients wore chest mounted, medical grade heart rate monitors during testing. Heart rates were recorded by a central CPU and continuously observed by study staff. After an initial 5-min warm-up period, power demands were increased every 3 min until one of the following conditions was reached: heart rate ≥ 180 bpm, patient could no longer turn the cranks, patient stood on the cranks, or patient reported exhaustion and a desire to stop. Assessments were performed at baseline (prior to any local or systemic therapy), 6 months, and 12 months after enrollment.

#### Body composition

A secondary objective was to explore the relationship of energetic capacity to body mass index (BMI) and body composition. Dual resistant X-ray absorptiometry (DEXA) was used to measure total body composition, including BMI, total lean body mass, appendicular lean mass, and percentage of body fat at baseline, 6 months, and 12 months following enrollment. Skeletal muscle index (SMI) was calculated as the appendicular lean mass in kilograms divided by height squared. Given its important association with reduced survival in several solid tumor populations [12], sarcopenia incidence was calculated for each cohort and defined as SMI two standard deviations below young adult female population norms (< 5.45 kg/m^2^) [13].

### Statistical analysis

This longitudinal cohort pilot study quantified the impact of breast cancer therapy on energetic capacity as measured by power generation. Sample size was determined by feasibility and precision around the estimates needed to design further studies. A minimum of 12 patients per group were needed; to account for drop-outs and missing data, and to increase precision around the estimates, we planned to enroll of up to 28 patients per cohort. The primary endpoints were changes in power per kilogram of lean body mass and mean peak watts over 10 s expended during the Power Protocol™ test between time points with T-tests to determine significance at the 0.05 level. Spearman’s correlations were used to evaluate the relationship between power and BMI, lean body mass, body fat percentage, and SMI.

## Results

### Patient characteristics

**Fig. 1:**
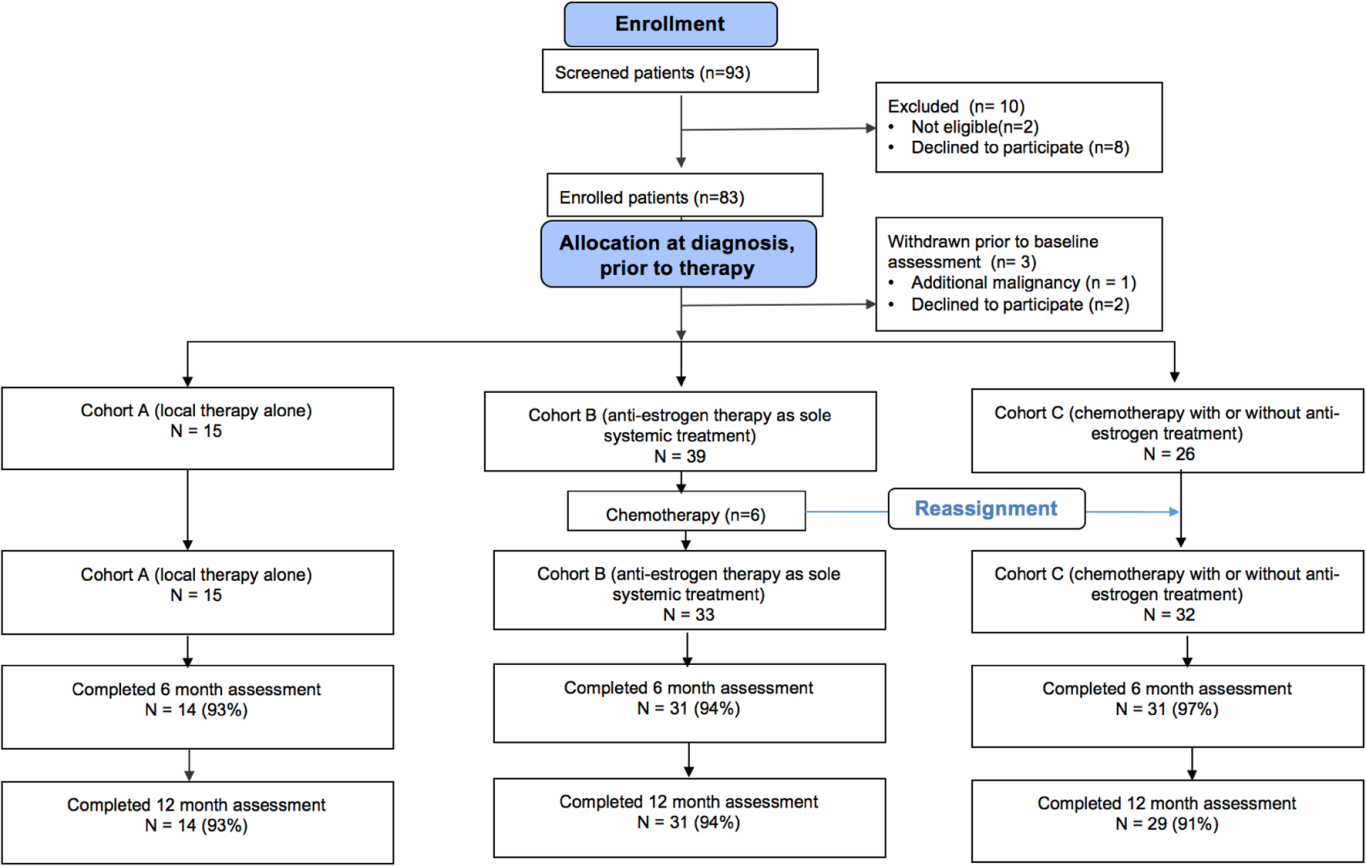
Consort diagram

A total of 83 eligible patients were enrolled (Fig. 1). One patient was found to have renal cell carcinoma and two others withdrew during screening prior to the baseline assessments. Eighty patients completed at least one assessment and thus were evaluable. Six patients initially enrolled to cohort A or B were transferred to cohort C when final pathology dictated the need for chemotherapy. Final analysis is based on 15 patients in cohort A, 33 in cohort B, and 32 in cohort C.

Four patients withdrew prior to the 6-month time point and 2 prior to the 12- month assessment. Overall, 74 patients (93%) completed all planned PowerProtocol™ assessments. All patients were female. Median age was 55; patients who received chemotherapy (cohort C) tended to be slightly younger. Most (44%) had stage I disease. Nearly half of patients receiving chemotherapy also received anti-estrogen treatment (Table 1).

**Table 1:**
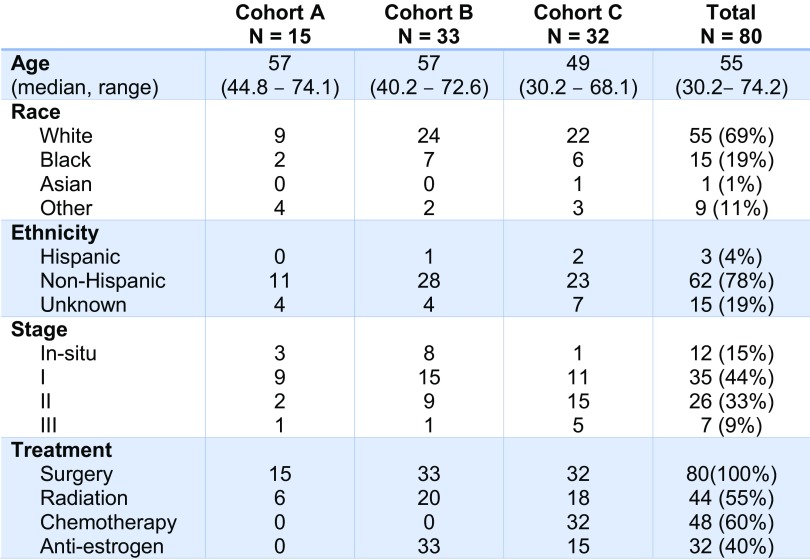
Patient demographics and disease characteristics of study sample

### Power generation

In the total study population, patients were deconditioned at baseline with power generation of 62.7 watts (SD 32.96 watts). There was no difference in power generation between the cohorts at baseline. The apparent higher level of baseline power in Cohort A (A: 74.9 watts, B: 56.5 watts, C: 63.0 watts) did not reach significance (*p* = 0.241). In the total population, power decreased at 6 months (62.7 vs. 48.2 watts; *p* < 0.001) and did not significantly recover at 12 months (48.2 vs. 49 watts; *p* = 0.978). At 6 months, power output dropped significantly in the cohorts receiving endocrine therapy (B: 56.5–42.11 watts, *p* = 0.003) and chemotherapy (C: 63.6–46.6 watts, *p* < 0.001), but not in patients receiving local therapy alone (A: 74.9–69.5 watts *p* = 0.06). In each cohort, there was no significant change from 6 to 12 months (A: + 3.4 watts, *p* = 0.6; B: + 1.4 watts, *p* = 0.97; C: − 1.4 watts, *p* = 0.76).

**Fig. 2:**
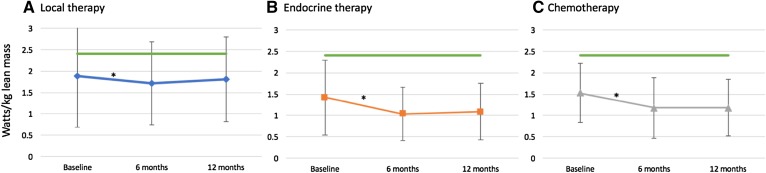
Power generation (watts, normalized to lean body mass) over time based on therapy received for early-stage breast cancer, **a** local therapy only; **b** endocrine therapy as the only systemic therapy; or **c** chemotherapy ± endocrine therapy. The green line represents a “functional norm” of power generation of 2.4 watts/kg lean body mass [14]. Power dropped significantly between baseline and 6 months in all cohorts (A: *p* = 0.04, B: *p* = 0.008, and C: *p* = < 0.001). * = *p* < 0.05

**Fig. 3:**
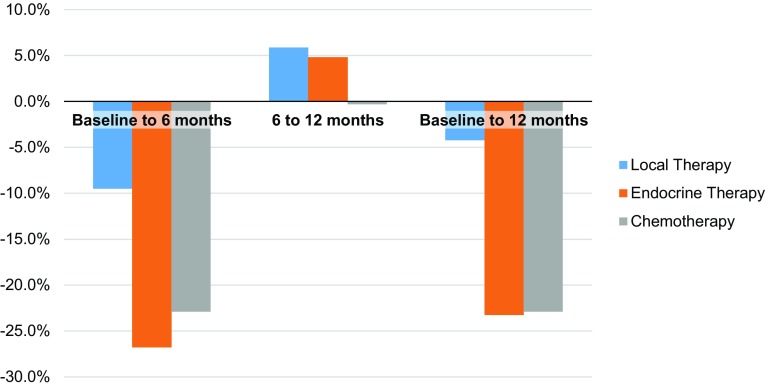
Percentage change in power normalized to lean body mass (watts/kg lean mass). Overall, from baseline to 12 months, mean power per kg of lean mass decreased by 4.2% in cohort A (*p* = 0.10), 23.2% in cohort B (*p* = 0.004), and 22.9% in cohort C (*p* < 0.001)

In all cohorts, power generation normalized to lean mass (watts/kg) dropped at 6 months (Fig. 2), most significantly in cohorts B and C (A: 1.89–1.71 watts/kg, *p* = 0.04; B: 1.42–1.04 watts/kg, *p* = 0.008; C: 1.53–1.18 watts/kg, *p* < 0.001). There was no significant change in power/kg lean mass between 6 and 12 months in any cohort (A: 1.71–1.81 watts/kg, *p* = 0.52; B: 1.04–1.09 watts/kg, *p* = 0.77; C: 1.18–1.18 watts/kg, *p* = 0.88). For the cohorts receiving systemic therapy, the decline in power/kg lean mass from baseline to 12 months (Fig. 3) was 19% in the endocrine therapy group (cohort B, *p* = 0.004) and 28% in the chemotherapy ± endocrine therapy group (cohort C, *p* < 0.001).

### Body composition

Body composition, including BMI, lean body mass, body fat percentage, and SMI were similar among cohorts at baseline (Table 2). Most patients were obese (BMI ≥ 30 kg/m^2^) at study entry and remained obese throughout the study period. Mean lean body mass declined significantly over 12 months in patients receiving chemotherapy ± anti-estrogen therapy (cohort C − 1.5 kg, *p* = 0.007). Body fat increased significantly in patients receiving anti-estrogen therapy alone (cohort B + 2.1%, *p* < 0.001) and in patients receiving chemotherapy ± anti-estrogen therapy (cohort C + 2.8%, *p* < 0.001), but was unchanged in patients receiving local therapy alone.

**Table 2:**
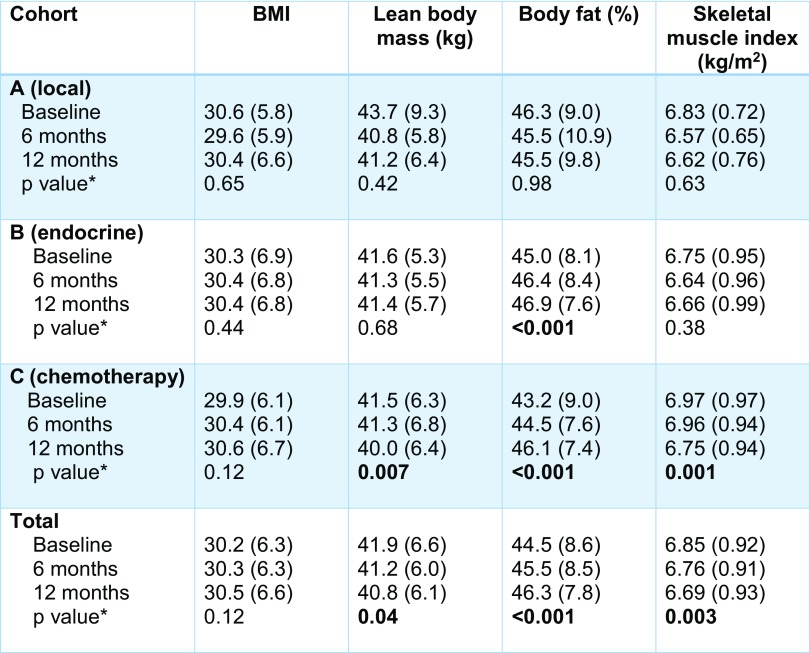
Body composition changes from diagnosis to 12 months among early-stage breast cancer patients receiving localized treatment only (A), endocrine therapy (B), or chemotherapy ± endocrine therapy (C)

*p* values that are significant (*p* < 0.05) are highlighted in bold

All values expressed as means with (standard deviation)

*p value describes change from baseline to 12 months, < 0.05 is significant

SMI (appendicular lean mass normalized to height) declined toward the sarcopenic range in all cohorts and significantly decreased in cohort C (mean difference − 0.33 kg, *p* = 0.001) throughout the study period. One patient in cohort A was sarcopenic at baseline and subsequently recovered. By 6 months, 4 patients in cohort B had developed sarcopenia, 2 of whom had persistent sarcopenia at 12 months. One patient in cohort C developed sarcopenia at 6 months with an additional 2 developing sarcopenia by 12 months.

### Correlation between power generation and body composition

Finally, we explored the relationship between power characteristics and changes in body composition. In the overall study population, we found a significant correlation between BMI and power at each time point (baseline *r* = − 0.25, *p* = 0.03; 6 months *r* = − 0.31, *p* = 0.01; 12 months *r* = − 0.34, *p* = 0.01). Similar findings were seen with body fat percentage. Interestingly, there was no correlation between change in power and BMI or SMI except for baseline to 6 months in cohort B only (Supplemental table 1).

## Discussion

Our study found an alarming rate of deconditioning in early-stage breast cancer patients at baseline, which only worsened following primary therapy and did not improve by 12 months after diagnosis. Although the post-treatment physical activity trajectories and fitness levels of breast cancer patients have been characterized in the control arms of exercise intervention studies, these suffer from significant selection bias, with the least fit patients unlikely to participate. Our results better reflect the larger clinical population as our patients were not involved in, or interested in, a lifestyle intervention [15]. Given the low levels of energetic capacity seen in our study, it is unlikely that this population would be able to comply with current physical activity recommendations. Importantly, our study also documents the variability in energetic capacity among our patients, highlighting the need for interventions to be personalized in order to help patients return to or improve upon pre- treatment fitness levels.

Several other studies examining the “fitness” of breast cancer survivors have used different assessments yet reached similar conclusions. Jones et al [16] evaluated VO_2_ max in patients before, during, and after adjuvant therapy, as well as in the metastatic setting. They found that breast cancer survivors have a VO_2_ max similar to someone 20–30 years older when compared to population normative data. In addition, one-third of patients had a VO_2_ max less than the threshold for functional independence. The Jones study was cross-sectional rather than prospective and did not examine the impact of the type of treatment received. Lakoski and colleagues examined the time to exhaustion on a treadmill in the Cooper Center Longitudinal Study [17], comparing the effect of adjuvant therapy in breast cancer patients to non-cancer age matched controls. They found that multimodality adjuvant therapy (surgery, radiation, and chemotherapy) significantly impairs fitness levels. However, in this cross-sectional study, patients were an average of 7 years from initial diagnosis at the time of evaluation. In addition, the effect of endocrine therapy was not assessed. Our study is the first to prospectively describe the power generation capabilities of early-stage breast cancer patients before and after primary therapy, and to evaluate these patients based on the treatment modalities received.

The reasons for poor baseline power generation abilities seen in our study are likely multi-factorial and vary between individual patients. It remains unclear if poor baseline function is a systemic effect of cancer prior to diagnosis; or whether patients who are debilitated, and presumably less active, are more likely to develop a clinically apparent cancer. Observational evidence suggests that more active individuals are less likely to be diagnosed with breast cancer [18, 19]. In our study, we did see a numerically higher baseline energetic capacity in the group of patients who required only local therapy (cohort A), the majority of whom had stage I and presumably less aggressive disease. While this result was not statistically significant, it is hypothesis generating.

While skeletal muscle wasting and/or muscle weakness has been appreciated in patients with advanced cancer or those receiving chemotherapy [12, 20, 21], this phenomenon is not well recognized in patients with early-stage disease and in those who do not receive chemotherapy. Unexpectedly, we saw a very similar trajectory of decline in function in patients treated with endocrine therapy alone as in those treated with chemotherapy. While endocrine-treated patients had a decline in function, there was no decline in lean body mass, suggesting true muscle dysfunction rather than muscle loss. Recent preclinical data have reported that maladaptive molecular changes occurring in skeletal muscle in response to an increased bone turnover/low estrogen state may be responsible for muscle dysfunction independent of loss in mass [22, 23].

Seven patients (8.8%) in our study developed frank sarcopenia during the 12 months following diagnosis. This incidence is lower than described in other studies; for example, the incidence of sarcopenia up to 12 months after diagnosis in a subset of the observational HEAL study of stage I–IIIA breast cancer patients was 15.9% [24]. This difference may be due to the inclusion of DCIS and a larger proportion of overweight and obese patients in our study. The HEAL study found sarcopenia to be an independent predictor of mortality, further supporting the need for interventions to address muscle loss and dysfunction in this population.

Our study has several limitations. First, results are limited by a small sample size and a lack of non-breast cancer controls. However, the data generated here can be compared to population norms and form the basis of future interventional studies. In addition, we did not attempt to isolate the effect of specific chemotherapy regimens (anthracyclines vs. not) or various endocrine therapies (aromatase inhibitor vs. selective estrogen receptor modulator). These deserve more detailed examination in the future.

In long-term follow-up of the Women’s Health Initiative Study, nearly 75% of post-menopausal women self-reported minimal physical activity (< 8 MET h/week) and activity levels remained stable over the 8 years of follow-up [25]. Together, these data and our results suggest that without intervention, the inactive and deconditioned breast cancer survivors (the majority of our population) are unlikely to improve. The alarming rate of deconditioning at baseline in all cohorts points toward the need for interventions in the prevention setting, and supports the possible role of “pre-habilitation” prior to, or in conjunction with, cancer-directed therapy. These findings also speak to the need for individualized pre- and post-treatment “oncologic rehabilitation” programs, similar to cardiac rehabilitation, as a crucial component of cancer survivorship care. To that end, we have begun to utilize baseline energy capacity parameters from the Power Protocol™ to design individualized physical activity recommendations in a currently active clinical trial (Individualized Metabolic Rx (iMETx), NCT03158519).

## Electronic supplementary material

Below is the link to the electronic supplementary material.

Supplementary material 1 (DOCX 14 KB)
