# Supplementary material for: Impact of primary breast cancer therapy on energetic capacity and body composition
Source: Breast Cancer Res Treat. 2018 Aug 22;172(2):445–52. doi: 10.1007/s10549-018-4924-6 (PMC6208924; doi:10.1007/s10549-018-4924-6)
Supplement: Supplementary file 1 — Supplementary material 1 (DOCX 14 KB) [file 10549_2018_4924_MOESM1_ESM.docx]

**Supplemental Table 1** Spearman’s Correlation with Power – n, correlation coefficient (p-value)

| **Variable** | **Treatment** | **Baseline** | **6 months** | **12 months** | **Change from baseline to 6 months** | **Change from baseline to 12 months** |
| --- | --- | --- | --- | --- | --- | --- |
| BMI (kg/m^2^) | Overall | 73, -0.254 (0.030) | 70, -0.311 (0.009) | 61, -0.344 (0.007) | 68, -0.221 (0.070) | 60, 0.007 (0.959) |
|  | Surgery alone | 13, -0.824 (<0.001) | 11, -0.482 (0.133) | 11, -0.327 (0.326) | 11, -0.132 (0.699) | 11, -0.064 (0.853) |
|  | Hormone alone | 30, -0.204 (0.280) | 30, -0.205 (0.277) | 28, -0.453 (0.016) | 28, -0.419 (0.027) | 27, -0.214 (0.283) |
|  | Chemo +/- Hormone | 30, -0.044 (0.817) | 29, -0.273 (0.152) | 22, -0.248 (0.266) | 29, -0.128 (0.508) | 22, 0.162 (0.471) |
|  |  |  |  |  |  |  |
| Lean body mass (kg) | Overall | 72, 0.041 (0.732) | 71, -0.168 (0.160) | 64, -0.080 (0.528) | 68, -0.130 (0.292) | 62, -0.021 (0.873) |
|  | Surgery alone | 13, -0.187 (0.541) | 11, -0.382 (0.247) | 11, -0.355 (0.285) | 11, 0.150 (0.659) | 11, 0.036 (0.916) |
|  | Hormone alone | 30, -0.040 (0.265) | 31, -0.154 (0.409) | 28, -0.323 (0.094) | 29, -0.314 (0.098) | 27, -0.057 (0.776) |
|  | Chemo +/- Hormone | 29, 0.0203 (0.290) | 29, -0.199 (0.301) | 25, 0.201 (0.336) | 28, -0.006 (0.977) | 24, -0.195 (0.362) |
|  |  |  |  |  |  |  |
| Body fat (%) | Overall | 72, -0.266 (0.024) | 70, -0.254 (0.034) | 64, -0.317 (0.011) | 67, -0.150 (0.225) | 62, 0.007 (0.954) |
|  | Surgery alone | 13, -0.846 (<0.001) | 11, -0.509 (0.110) | 11, -0.382 (0.247) | 11, 0.068 (0.841) | 11, 0.000 (1.000) |
|  | Hormone alone | 30, -0.040 (0.833) | 31, -0.210 (0.257) | 28, -0.295 (0.128) | 29, -0.200 (0.297) | 27, -0.156 (0.441) |
|  | Chemo +/- Hormone | 29, -0.093 (0.632) | 28, -0.182 (0.354) | 25, -0.217 (0.297) | 27, -0.269 (0.174) | 24, 0.200 (0.350) |
|  |  |  |  |  |  |  |
| Skeletal muscle index (kg/m^2^) | Overall | 73, 0.124 (0.297) | 70, -0.035 (0.772) | 59, -0.147 (0.267) | 68, -0.040 (0.745) | 58, 0.156 (0.242) |
|  | Surgery alone | 13, -0.165 (0.591) | 11, -0.264 (0.433) | 10, -0.067 (0.855) | 11, -0.323 (0.332) | 10, -0.273 (0.446) |
|  | Hormone alone | 30, 0.040 (0.835) | 30, -0.007 (0.971) | 27, -0.351 (0.073) | 28, -0.386 (0.042) | 26, 0.035 (0.866) |
|  | Chemo +/- Hormone | 30, 0.296 (0.112) | 29, -0.087 (0.655) | 22, -0.023 (0.919) | 29, 0.277 (0.146) | 22, 0.275 (0.216) |
